# Data Mining and Content Analysis of the Chinese Social Media Platform Weibo During the Early COVID-19 Outbreak: Retrospective Observational Infoveillance Study

**DOI:** 10.2196/18700

**Published:** 2020-04-21

**Authors:** Jiawei Li, Qing Xu, Raphael Cuomo, Vidya Purushothaman, Tim Mackey

**Affiliations:** 1 Department of Anesthesiology and Division of Infectious Diseases and Global Public Health University of California San Diego School of Medicine La Jolla, CA United States; 2 S-3 Research LLC San Diego, CA United States; 3 Department of Healthcare Research and Policy University of California San Diego Extension La Jolla, CA United States; 4 Global Health Policy Institute San Diego, CA United States

**Keywords:** COVID-19, coronavirus, infectious disease, social media, surveillance, infoveillance, infodemiology

## Abstract

**Background:**

The coronavirus disease (COVID-19) pandemic, which began in Wuhan, China in December 2019, is rapidly spreading worldwide with over 1.9 million cases as of mid-April 2020. Infoveillance approaches using social media can help characterize disease distribution and public knowledge, attitudes, and behaviors critical to the early stages of an outbreak.

**Objective:**

The aim of this study is to conduct a quantitative and qualitative assessment of Chinese social media posts originating in Wuhan City on the Chinese microblogging platform Weibo during the early stages of the COVID-19 outbreak.

**Methods:**

Chinese-language messages from Wuhan were collected for 39 days between December 23, 2019, and January 30, 2020, on Weibo. For quantitative analysis, the total daily cases of COVID-19 in Wuhan were obtained from the Chinese National Health Commission, and a linear regression model was used to determine if Weibo COVID-19 posts were predictive of the number of cases reported. Qualitative content analysis and an inductive manual coding approach were used to identify parent classifications of news and user-generated COVID-19 topics.

**Results:**

A total of 115,299 Weibo posts were collected during the study time frame consisting of an average of 2956 posts per day (minimum 0, maximum 13,587). Quantitative analysis found a positive correlation between the number of Weibo posts and the number of reported cases from Wuhan, with approximately 10 more COVID-19 cases per 40 social media posts (*P*<.001). This effect size was also larger than what was observed for the rest of China excluding Hubei Province (where Wuhan is the capital city) and held when comparing the number of Weibo posts to the incidence proportion of cases in Hubei Province. Qualitative analysis of 11,893 posts during the first 21 days of the study period with COVID-19-related posts uncovered four parent classifications including Weibo discussions about the causative agent of the disease, changing epidemiological characteristics of the outbreak, public reaction to outbreak control and response measures, and other topics. Generally, these themes also exhibited public uncertainty and changing knowledge and attitudes about COVID-19, including posts exhibiting both protective and higher-risk behaviors.

**Conclusions:**

The results of this study provide initial insight into the origins of the COVID-19 outbreak based on quantitative and qualitative analysis of Chinese social media data at the initial epicenter in Wuhan City. Future studies should continue to explore the utility of social media data to predict COVID-19 disease severity, measure public reaction and behavior, and evaluate effectiveness of outbreak communication.

## Introduction

The coronavirus disease (COVID-19) is a rapidly emerging infectious disease caused by a novel coronavirus named severe acute respiratory syndrome (SARS) coronavirus 2 [1]. The COVID-19 outbreak began in late December 2019 in Wuhan, Hubei Province, China, with a cluster of patients presenting with pneumonia of unknown origin and reported exposure to a seafood and live animal market in the same city [2]. The World Health Organization (WHO) confirmed 41 cases and 1 death due to the novel coronavirus by January 12, 2020 [3]. Since this initial reporting, COVID-19 has rapidly spread within China and internationally, with the WHO declaring a Public Health Emergency of International Concern (PHEIC) under the revised International Health Regulations on January 30, 2020 [4].

Since the PHEIC declaration, COVID-19 has spread to every continent except Antarctica, becoming a highly infectious global pandemic with sustained community transmission [5]. The severity of the COVID-19 outbreak, with approximately 1.8 million cases worldwide as of mid-April 2020 [6], has far surpassed past coronavirus events such as the Middle East respiratory syndrome (MERS)–related coronavirus, which had 2494 cases as of November 2019, and the 2003 SARS coronavirus, which had more than 8000 cases and affected 26 countries. It is unknown whether viral mutations will result in patterns of annual re-emergence as seen with influenza strains.

Attempts to predict epidemiological features (eg, prevalence, attack rate, replication or reproduction rate, morbidity, and mortality) of an outbreak to inform infection control and public health countermeasures are critical. This can be challenging during the earlier stages of an outbreak when there is a lack of sufficient information regarding the etiology of the disease, inadequate diagnostic and testing capabilities, and incomplete epidemiological data regarding confirmed cases [7]. In the absence of such data, the use of information in an electronic medium such as social media conversations can enable syndromic surveillance approaches to characterize disease distribution and provide accurate case counts more rapidly [8].

These “infoveillance” approaches have been used to characterize a host of public health issues including topics related to mental health, substance abuse behavior, the spread of foodborne illness, and the monitoring of infectious disease outbreaks (eg pertussis, influenza, HIV/AIDS, dengue, West Nile virus, Zika virus, H1N1, and Ebola) [8-12]. Specifically, the now ubiquitous nature of social media means it represents an important, “nontraditional” source for disease surveillance. Specifically, user-generated social media data can be mined to assess the public’s knowledge, attitudes, and behaviors toward the disease, and can be particularly informative when cross-validated with traditional disease surveillance data [13-17]. Others have also used global social media platforms such as Twitter to examine the 2009 H1N1 pandemic, conduct content analysis, and identify key trends that may also correlate with outbreak incidence data [18].

Leveraging infoveillance approaches, we conducted a retrospective observational study for COVID-19 on one of the largest Chinese social media platforms, Sina Weibo [新浪微博]. Sina Weibo is a microblogging website (also known as the Chinese equivalent to Twitter) and one of the most influential social media platforms in China. According to its own press release in August 2020, it had over 486 million active users [19]. Users can publish content such as messages in microblogs and share text, pictures, videos, and music. Compared with WeChat, another popular social media platform in China, Weibo posts are generally more publicly visible; with WeChat, posts are generally more private and only visible to certain people selected by users. Due to the public nature of the platform, we attempted to assess whether Weibo posts about COVID-19 were predictive of the number of reported cases during the outbreak’s early stages and conducted a qualitative analysis of COVID-19–related themes detected and discussed by users located in Wuhan.

## Methods

### Study Design

This observational infoveillance study was conducted in two phases: data collection using an automated Python (Python Software Foundation) programming script to collect COVID-19-related posts on Weibo, and quantitative and qualitative analysis to identify trends and characterize key themes discussed by Chinese users.

### Data Collection

Programming scripts were written in the Python programming language to extract posts on Weibo in the Chinese language (traditional and simplified Chinese) from users self-reporting their location in Wuhan. Weibo users can post messages limited to 2000 characters with or without images, videos, and other multimedia, and can repost messages equivalent to the retweet function on Twitter. Python scripts were set to continuously collect data filtered for COVID-19–related keywords from December 23, 2019, to January 30, 2020. Keywords included the Chinese-language terms: [冠状病毒] (coronavirus), [新型肺炎] (novel pneumonia), [武汉肺炎] (Wuhan pneumonia), [疫情] (epidemic situation), [非典] (severe acute respiratory syndrome), [华南海鲜市场] (Wuhan Seafood Wholesale Market). These keywords were chosen on the basis of manual searches via the platform’s public search function to detect a baseline of user conversations related to the outbreak for our systematic data collection processes. The variation in selected keywords was also necessary, as the official name of the disease was not announced until February 11, 2020 [20]. These data collection methods are consistent with other analyses of health-related posts (including flu-related topics) on the Weibo platform not related to COVID-19 [21].

Posts were filtered for geographic location, thereby identifying users specifically within the city of Wuhan, China. Posts with geographic locations outside of Wuhan City were not included in this study, as the aim was to focus on the early origins of the outbreak in this region. Posts were collected from all account types including personal accounts, media accounts, and government accounts. Technical limitations of the Weibo platform and Python script used to collect data limited our data collection to a maximum of 2000 posts per hour. However, during the 936 hours of data collection, we did not reach this limit for any hour of collection.

The number of COVID-19 cases in all of mainland China and Hubei Province were collected for each calendar day between December 23, 2019, and January 30, 2020, inclusively. Case counts were made publicly available on the internet by the Chinese National Health Commission, a cabinet-level executive department of the Chinese central government, headquartered in Beijing [20].

### Quantitative Analysis

Weibo posts with COVID-19-related keywords were binned into each calendar day to calculate posts per day. Longitudinal trends were then visually conveyed using line graphs. Regression analysis was conducted to understand the predictive value of social media posts on the number of confirmed cases reported by the Chinese government. Simple linear regression was performed between social media posts per day and the number of cases reported within mainland China, excluding Hubei Province, and the cases in Hubei Province alone. Also, a simple linear regression model was computed wherein the number of posts per day was used to predict percent daily change in cases from Hubei, and a separate model was computed to predict percent daily change in cases from mainland China (excluding Hubei). Modeling of daily changes was conducted using the final 20 days of data, as prior days did not exhibit daily changes in posts, cases from Hubei, or cases from the remainder of China. A *P* value <.05 was considered statistically significant for all analyses. Statistical analyses were performed using RStudio, version 3.6.1 (RStudio, Inc).

### Qualitative Analysis

Qualitative content analysis was conducted on the posts collected from December 31, 2019, to January 20, 2020, for key COVID-19-related themes self-reported by Chinese users and for information posted by the media and government sources.

Content analysis focused on detection of themes associated with knowledge, beliefs, and health behaviors specifically related to COVID-19 topics. Reviewers examined a random selection of Weibo posts with stratification of time periods so as to be representative of the entire period encompassed by the 39-day data collection.

First, coders independently used a binary coding approach (ie, relevant vs nonrelevant) to filter posts related to COVID-19 conversations and news, and exclude other “noise” not related to COVID-19. Second, we used thematic content analysis coding methods by first examining the meaning of words and their sentence structure in the text of Chinese-language Weibo posts. Third, we identified parent classifications to select prevalent topics and then tagged and grouped these classifications with supporting qualitative data (eg, Weibo posts). We primarily relied on inductive coding approaches starting with Weibo COVID-19 posts identified but also informed this inductive coding based on themes detected in existing literature from prior disease outbreaks [18,21]. Coders individually selected parent topic classifications to represent different thematic areas and collapsed infrequent categories into parent classifications. We then combined the related topics, removed duplicate topics, and evaluated thematic concurrence by independently coding the entire sample of posts collected from the early period of the outbreak with detected COVID-19-related posts (December 31, 2019, until January 20, 2020.)

## Results

### Data Availability and Ethics Approval

Data collected on social media platforms is available on request from authors subject to appropriate deidentification. Ethics approval was not required for this study. All information collected from this study was from the public domain, and the study did not involve any interaction with users. Indefinable user information was removed from the study results.

### Data Collection

There were 115,299 posts collected during the study time frame, with an average of 2956 Weibo posts per day. There was a high degree of variation in the number of posts depending on the date of collection, with 0 posts collected on one day (December 26, 2019) and the highest number of posts (13,740) collected on January 27, 2020. During this same time period, China reported 36,456 confirmed cases of COVID-19. COVID-19 cases were reported starting on January 16, 2020, when 45 cases were reported. The number of cases then increased rapidly, reaching 9692 cases on January 30, 2020, the final day of data collection for this study. For every 1 hour of data collection the average yielded posts were 128, this was much higher after official case estimates began to be reported by the Chinese government (an average of 314 posts per hour) than before reporting began (an average of 11 posts per hour). Hourly postings exhibited diurnal fluctuations, which corresponded to customary waking hours.

### Quantitative Analysis

The linear regression showed a positive relationship between Weibo posts and the number of cases officially reported in Hubei Province, with approximately 10 more COVID-19 cases per 40 social media posts (cases=0.242*posts–201.48; *P*<.001; *R*^2^=0.621). For the official number of cases within mainland China excluding Hubei Province, we found approximately 10 more COVID-19 cases per 60 social media posts (cases=0.164*posts–143.21; *P*<.001; *R*^2^=0.652). These results indicate that there was a statistically significant positive relationship with Weibo posts and official case counts from within Hubei, and that the effect size was larger than what was observed in the rest of mainland China excluding Hubei. The linear regression also showed a significant inverse relationship between Weibo posts and the incidence proportion of cases reported in Hubei among all incident cases in mainland China (Wuhan cases proportion=–0.003*posts+100.2; *P*<.001; *R*^2^=0.836). Social media posts were also predictive of the percentage increase in COVID-19 cases in mainland China excluding Hubei (percent daily case increase=0.00013*posts+0.02; *P*<.001; *R*^2^=0.827), but not Hubei alone (percent daily case increase=0.000016*posts+1.22; *P*=.23; *R*^2^=0.091). These results may imply that 1000 additional posts from Wuhan predicted a 13% day-to-day increase in the number of cases for the rest of China but did not predict a local increase in cases. Therefore, it is possible that the numbers of outbreak-related Weibo posts are reactive to local disease conditions while being predictive of disease conditions for the broader region.

**Figure 1:**
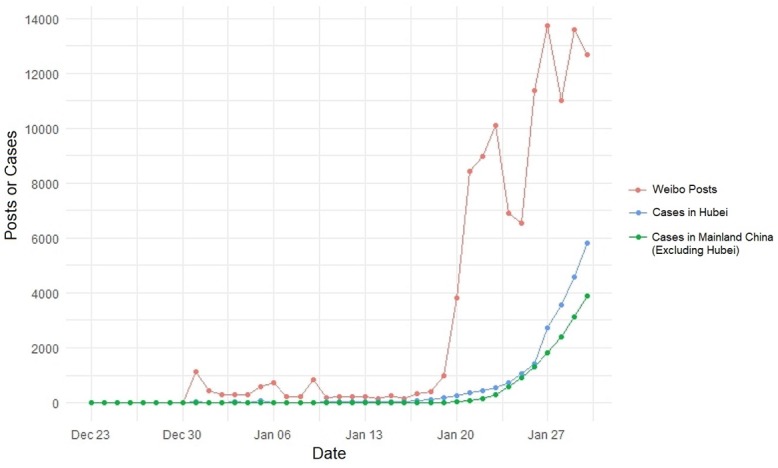
Longitudinal trends of Weibo coronavirus disease posts and coronavirus disease official case counts.

Visualization of longitudinal trends found that this association was generally uninterrupted, except for dramatically fewer posts on January 25 and January 28. The decreases for January 25 coincided with Chinese New Year celebrations, but this may not explain the decreases observed on January 28 (see Figure 1). Further observations in our qualitative analysis identified specific events and news during the study period that may have also influenced the number of user posts on certain observed dates and are further described in later sections.

### Qualitative Analysis

A total of 11,893 posts from the first 21 days of our study (December 31, 2019, to January 20, 2020) that detected COVID-19 posts were manually coded. Qualitative analysis revealed that certain common terms or language within the corpus of collected Weibo posts were reflective of the parent classification of early COVID-19 themes including “pneumonia of unknown cause” [不明原因肺炎], “Wuhan pneumonia” [武汉肺炎], “unknown virus pneumonia” [不明病毒肺炎], and “novel coronavirus” [新型冠状病毒]. The distribution of these terms varied during the study period, with “unknown virus pneumonia” returning the largest proportion of Weibo posts during the early outbreak period until January 9, 2020, after which the term “Wuhan coronavirus” began to generate more Weibo post mentions. This is likely due to the lack of a defined name for the disease early in the outbreak, which also led to confusion about whether a novel outbreak was occurring or if it was a re-emergence of SARS.

Several important themes were identified in our inductive content analysis of Weibo posts, including four parent classifications with discussions about the causative agent of the disease, the changing epidemiological characteristics of the outbreak, and the public reaction to outbreak control and response measures (see Table 1). Authors JL and QX manually annotated posts for the previously mentioned parent classification themes detected. For inconsistent results, authors met and reviewed the posts together and conferred on the correct classification. After manually annotating the early outbreak period data (December 31, 2019, to January 20, 2020), intercoder kappa agreement scores for each theme were as follows: the causative agent of the disease was 99.04%, the changing epidemiological characteristics of the outbreak was 98.26%, and the public reaction to the outbreak control and response measures was 97.27%.

A prevailing theme throughout the outbreak period that changed based on the availability of new information was that the causative agent of the outbreak was unknown, leading to uncertainty among Chinese users regarding the risks associated with the outbreak. This period of initial uncertainty was followed by the information disclosure about the causative agent by the Chinese National Health Commission and other official government and academic sources. As the outbreak progressed, a higher volume of more precise terms including “novel coronavirus” or “COVID-19” were detected, with a decline and shift from posts mentioning colloquial terms such as “unknown reason pneumonia” and “Wuhan pneumonia,” similar to terminology adoption during the H1N1 pandemic [18].

The presence of parent classifications also changed over the time period. For example, at the onset of the outbreak, many users discussed what the causative agent of the outbreak might be, including how seasonal influenza, avian influenza, MERS, and SARS were ruled out. From January 16-20, posts about the causative agent also increased with most conversations discussing a novel coronavirus strain. There were also early conversations about the South China Seafood Market and wildlife trafficking, reflecting uncertainty regarding the zoonotic origins and possible transmission vectors of the disease. The proportion of posts with reference to the South China Seafood Market were much higher prior to January 6.

The detection of posts related to the epidemiological characteristics of the outbreak were relatively consistent throughout the entire 21-day period assessed. However, following confirmation of the outbreak as a novel coronavirus, there was a spike of discussions about whether COVID-19 was transmittable human-to-human. Relatedly, at the beginning of the outbreak, there were some posts where users expressed their own personal reaction and concerns about a potential outbreak. From January 14, after 3 cases were confirmed outside of China, there was a notable increase in the number of posts related to the public's reaction to the outbreak and its associated risks and control and response measures.

More specific subthemes regarding users’ knowledge, attitudes, and responses to COVID-19 changed as more information about the underlining epidemiology became available. Specifically, the terms “confirmed case,” “suspected case,” “death case,” “human-to-human transmission,” “monitored,” “public health supervision,” and “quarantine” became more frequent as the outbreak progressed. Accompanying this shift in terminology, we also observed wide variation in user reactions as information from government sources was disseminated and the outbreak worsened. This included posts conveying protective behaviors (eg, cleaning hands, staying away from crowds, wearing medical masks in public areas), while others conveyed attitudes and behaviors that could potentially increase the risk of transmission (eg, going to New Year celebration events and self-evacuation from Wuhan). New user-response topics began to emerge toward the end of the early outbreak period, including criticism of the Wuhan Red Cross response and user uncertainty related to news about quarantines, travel restrictions, and new hospital construction projects.

Another subtheme detected in user responses was the discussion about COVID-19-related symptoms that appeared in both news and individual posts. At the beginning of the outbreak period, most symptom-related posts were posted by official accounts or consisted of news posts that were reposted and shared by the public that included describing symptoms of fever and shortness of breath (January 1) and coughing and weakness or fatigue (January 10). Separate from these news-related posts, some individual accounts also self-reported other symptoms including headache, diarrhea, sore throat, and perspiration during sleep. After January 20, 2020, human-to-human transmission was confirmed, and we detected an increase in posts related to user self-reported symptoms along with posts that provided second-hand reporting of symptoms from other people during our coding of the random stratified sample of the entire 39-day data study period. Though not fully coded for this study, our random selection detected a few users who reported other disputed symptoms, including a loss of taste and lack of appetite, in the last 10 days of our overall data collection (January 21-30).

Importantly, both the nature of the content and the volume of posts were likely driven by a combination of release of government information and news events. For example, December 31, 2019 had the second highest volume of posts related to the COVID-19 term “pneumonia of unknown cause,” which corresponded to the confirmation from an official source (the Wuhan Health Commission) of cases of pneumonia of unknown etiology detected in Wuhan city. There was also an increase in COVID-19 Weibo posts on January 6, 2020, likely driven by news on January 5 that laboratory test results had ruled out other pathogens (eg, influenza, avian influenza, adenovirus, MERS, and SARS) as the cause of the outbreak. Additionally, on January 15, the WHO announced that the possibility of limited human-to-human transmission could not be excluded. This drove a second increase in the overall number of posts on January 16. Finally, on January 20, human-to-human transmission was confirmed, generating a large number of conversations from both media accounts and private Weibo users, resulting in the largest increase of posts observed during this time period.

**Table 1:** Posts on the Weibo social media platform organized according to themes detected in qualitative analysis.

Theme, Description of topic (English)		Example topics (Chinese with English translation)
**Causative agent**		
	Concerns about potential SARS^a^ re-emergence	[希望不是非典，大家安心过年] “I wish this is not SARS, hope everyone can have a peaceful New Year Eve”
	Wuhan Seafood Wholesale Market considered the source of causative agent	[华南海鲜市场可能是病毒来源] “Wuhan Seafood Wholesale Market might be the source of the causative agent”
	Pneumonia caused by unknown reason	[武汉出现不明原因肺炎] “cases of pneumonia of unknown etiology detected in Wuhan City”
	Novel coronavirus has been confirmed as causative agent	[初步认定为新型冠状病毒] “Causative agent preliminary identification of a novel coronavirus”
**Epidemiological characteristics of the outbreak**		
	No evidence of human-to-human transmission	[目前没有证据证明人传人] “There is no evidence of human-to-human transmission”
	New confirmed cases and statistics on mortality	[截至昨日病例已增至44例，其中11例重症，均接受隔离治疗] “Until yesterday, there were 44 confirm cases, 11 severe cases, all have been isolated and treated”
	Public Health supervision for people who had close contact with patients with confirmed cases	[与病患接触者进行医学观察] “People who have close connection with confirmed cases are under medical supervision”
**Public reaction to outbreak control and response**		
	Wear masks, keep away from crowds	[虽然不知道是不是人传人，还是戴上口罩，远离人群] “Not sure if it is human to human transmissible, but wear masks and keep away from crowds would help. Just in case.”
	Self-evacuating Wuhan	[太可怕了，快点离开这儿吧] “It’s scary, let’s leave here (Wuhan)”
	Will still attend New Year celebration event	[不明原因肺炎丝毫不影响大家戴口罩出来跨年] “The pneumonia of unknown cause seems not to impact people’s New Year Eve celebration event”
**Other topics**		
	Wuhan Red Cross under scrutiny	[红十字会遭到社会指责] “The Red Cross was accused by society”
	Travel restrictions	[从1月23日10时起，武汉市和周边的鄂州市、仙桃市、潜江市、黄冈市、荆门市等相续宣布暂停运营城市公交、地铁、轮渡、长途客运，暂时关闭机场、火车站、高速公路等离开通道，严防武汉新型冠状病毒疫情扩散。] “From 10:00 on January 23, Wuhan and surrounding Ezhou, Xiantao, Qianjiang, Huanggang, Jingmen, etc. have successively announced the suspension of operation of city buses, subways, ferries, long-distance passenger transport, and temporarily closed airports and trains. Stations, highways, etc. leave the passage to strictly prevent the spread of new coronavirus epidemics in Wuhan.”
	New hospital construction projects	[新建武汉“小汤山] “Will build new hospitals in Wuhan (Lei Shen Shan & Huo Shen Shan), also called Wuhan Xiao Tang Shan”

^a^SARS: severe acute respiratory syndrome.

## Discussion

### Principal Findings

The vast majority of infoveillance studies have analyzed data from English-language social media platforms such as the microblogging site Twitter or Google trends data, yet only a few have examined foreign-language platforms. Due to the COVID-19 outbreak originating in Wuhan, China, this study sought to identify, characterize, and assess the potential relationship between Chinese social media conversations taking place in Wuhan and the number of COVID-19 confirmed cases at the early stages of the outbreak, which has now transitioned into a worldwide pandemic. It also sought to understand how user perception changed as additional information became available from government and media sources as the outbreak progressed while also attempting to identify parent classifications of predominant user-generated themes that emerged as the outbreak accelerated. These study objectives and some of its general findings are consistent with prior studies, including a 2010 infoveillance study by Chew and Eysenbach [18] assessing the 2009 H1N1 outbreak. In that study, posts from Twitter were collected, thematically assessed, and found to be significantly correlated with weekly H1N1 incidence during the outbreak, with the absolute increase in H1N1-related tweet volume coinciding with major news events.

Based on our analysis, there appears to be a positive correlation between the number of COVID-19-related Weibo posts from Wuhan and the number of cases officially reported in Wuhan during the early stages of this outbreak. This effect size was larger than what was observed for the rest of China excluding Hubei Province (where Wuhan is the capital city) and held when comparing the number of Weibo posts to the incidence proportion of cases in Hubei. However, any potential predictive value of using social media data as a proxy for real world public health surveillance statistics needs more rigor and added data layers to confirm possible associations, particularly in the context of user reactions to news events as discussed in this and other studies. Despite these limitations, qualitative analysis characterized the early stages of the outbreak as having different degrees of the Chinese public’s uncertainty regarding the risks posed by COVID-19. As information emerged about the disease, users expressed new concerns, which also led to changing knowledge, attitudes, and behaviors among Chinese social media users, some protective and some that may have introduced increased risks of disease spread.

**Figure 2:**
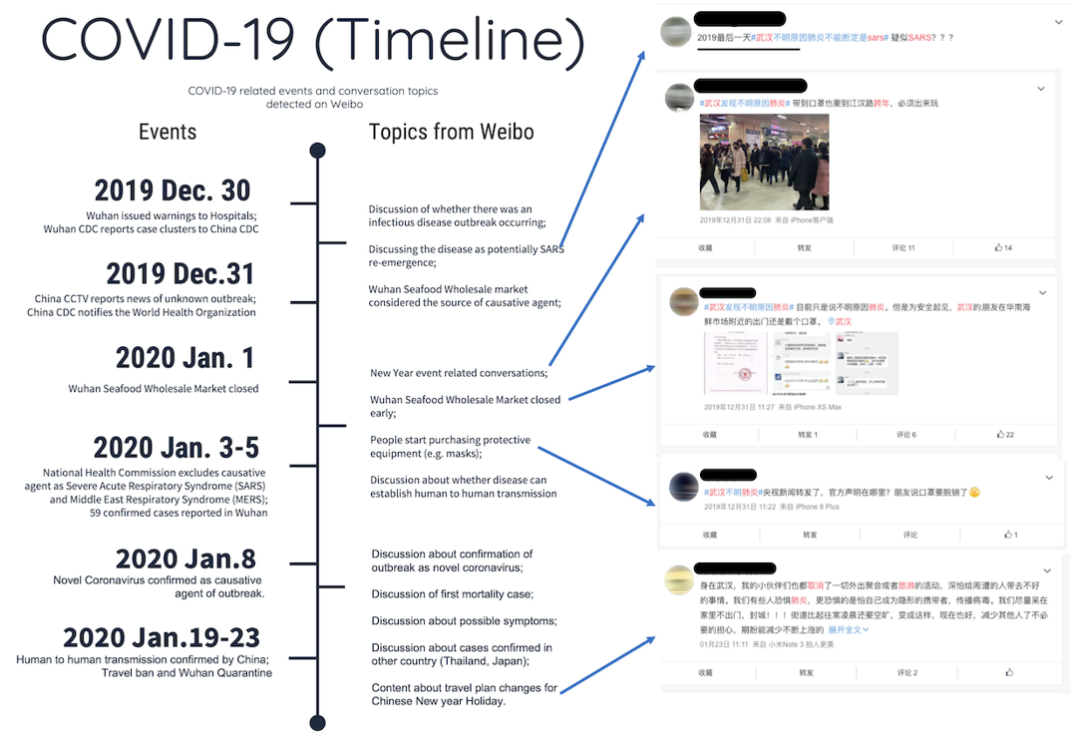
Timeline of COVID-19 events, themes detected in Weibo posts, and examples of posts. CCTV: China Central Television; CDC: Centers for Disease Control and Prevention; COVID-19: coronavirus disease.

In response to public uncertainty, the Chinese government issued a series of announcements on the Weibo “official” accounts in an attempt to clarify the characteristics of the disease as they became known, including an official warning to hospitals on December 30, 2019, about how to report potential cases and a subsequent announcement on January 8, 2020, confirming a novel coronavirus as the causative disease agent (Figure 2). These events evidence that social media was used as an outbreak communication tool by the government, media, and users (who reposted content) and led to the dissemination of and user reaction to information on an outbreak whose trajectory would take it global.

Public perception about the origins and transmission patterns of COVID-19 changed over the study period, with initial conversations focusing on a possible link with the seafood market in Wuhan, later changing to discussions about the increased number of cases reporting no exposure to the seafood market, and then leading to discussions of possible human-to-human transmission, which was later confirmed by the Chinese government. Overall, we observed a wide variation in user reactions to information, with some users expressing a willingness to undertake protective behavior and other users downplaying the risks and engaging in behaviors that could have exacerbated disease spread (eg, leaving Wuhan, attending New Year events). Importantly, these observed attitudes and behaviors happened prior to the Chinese government announcing a lockdown of Wuhan and other cities in Hubei on January 23, 2020. After the announcement of these quarantine measures, there was a sizable increase in the volume of Wuhan Weibo posts we collected.

Though the mixed quantitative and qualitative results of this study are primarily exploratory, they provide important insight on the changing knowledge, attitudes, and behaviors of Chinese social media users who were at the epicenter of what is now a rapidly expanding pandemic that has impacted all facets of global society. More research is needed to better understand the effectiveness of health communication strategies during evolving outbreaks such as COVID-19, particularly in the context of how information is understood, shared, and acted upon by users in the face of uncertainty and changing information. Specifically, we need to better understand how social media platforms can influence the public’s risk perception, their trust and credibility of different information sources, and, ultimately, how it changes real-world behavior that can have an impact on control measures enacted to mitigate an outbreak.

Early reports indicated that major social media platforms are struggling with the volume of COVID-19 information and user-generated content flooding their platforms, some of which is helpful and accurate and some of which are rumors and misinformation [22]. In fact, popular social media platforms TikTok, Facebook, and Twitter have all announced measures to better ensure access to credible and accurate information about COVID-19, though whether these platforms are up to this task remains an open question [22].Evaluating whether social media can act as a positive tool to promote global health objectives, particularly in the context of health emergencies, will be tested by COVID-19, along with its utility as a modern approach to public health surveillance.

### Limitations

This study has certain limitations. First, our data collection was limited to one Chinese social media platform over a prescribed period of time. Hence, it is not generalizable to all COVID-19 social media conversations occurring among Chinese users. We did not examine Chinese private communication apps (eg QQ, WeChat) due to the difficulty of collecting data on these platforms. Future studies should assess a broader scope of conversational data from multiple platforms and use natural language processing and machine learning approaches to help classify larger volumes of conversations. Second, our data collection started from the reported early stages of the COVID-19 outbreak. During parts of this time period, the causative agent had not been confirmed and there was no official name for the disease. Due to early inconsistency in terminology, Weibo users may have used other keywords to describe COVID-19 that were not collected in this study. Third, the simple linear regression showed a positive correlation between COVID-19 Weibo posts and the number of Wuhan cases in the study time frame, but we did not control for other potential confounders. Further, it is unclear whether our observed predictive trend line would continue or if the trend line is generalizable to other instances of COVID-19 outbreaks in other countries or communities. It is more likely that only under specific disease transmission circumstances would this correlation occur, namely a lack of knowledge and reporting of an outbreak in its early stages, a novel virus with high transmission and sustained community spread, and high social media engagement involving outbreak conversations. Further, as previously stated, it is highly likely that the volume of posts are associated with user reactions to news events and government announcements. Finally, due to censorship in China, posts may have been deleted before data collection. In fact, a few messages detected included associated comments that had been deleted and were not retrievable.

## Abbreviations

COVID-19: coronavirus disease
MERS: Middle East respiratory syndrome
PHEIC: Public Health Emergency of International Concern
SARS: severe acute respiratory syndrome
WHO: World Health Organization
